# Catatonia and elevated mortality: A population‐wide cohort study with healthy, sibling, and schizophrenia spectrum controls

**DOI:** 10.1111/pcn.13915

**Published:** 2025-11-13

**Authors:** Chih‐Wei Hsu, Yang‐Chieh Brian Chen, Marco Solmi, Chih‐Sung Liang, Mu‐Hong Chen, Yao‐Hsu Yang, Liang‐Jen Wang, Edward Chia‐Cheng Lai

**Affiliations:** ^1^ Department of Psychiatry Kaohsiung Chang Gung Memorial Hospital and Chang Gung University College of Medicine Kaohsiung Taiwan; ^2^ Department of Psychiatry and Behavioral Sciences The University of Texas Health Science Center at Houston Houston Texas USA; ^3^ Department of Child and Adolescent Psychiatry Charité Universitätsmedizin Berlin Germany; ^4^ Department of Psychiatry University of Ottawa Ottawa Ontario Canada; ^5^ Department of Mental Health The Ottawa Hospital Ottawa Ontario Canada; ^6^ Ottawa Hospital Research Institute Ottawa Ontario Canada; ^7^ School of Epidemiology and Public Health, Faculty of Medicine University of Ottawa Ottawa Ontario Canada; ^8^ Department of Psychiatry, Beitou branch, Tri‐Service General Hospital National Defense Medical University Taipei Taiwan; ^9^ Department of Psychiatry National Defense Medical University Taipei Taiwan; ^10^ Department of Psychiatry Taipei Veterans General Hospital Taipei Taiwan; ^11^ Department of Psychiatry, College of Medicine National Yang Ming Chiao Tung University Taipei Taiwan; ^12^ Department of Traditional Chinese Medicine Chiayi Chang Gung Memorial Hospital Chiayi Taiwan; ^13^ Health Information and Epidemiology Laboratory of Chang Gung Memorial Hospital Chiayi Taiwan; ^14^ School of Traditional Chinese Medicine, College of Medicine Chang Gung University Taoyuan Taiwan; ^15^ Department of Child and Adolescent Psychiatry, Kaohsiung Chang Gung Memorial Hospital Chang Gung University College of Medicine Kaohsiung Taiwan; ^16^ School of Pharmacy, Institute of Clinical Pharmacy and Pharmaceutical Sciences, College of Medicine National Cheng Kung University Tainan Taiwan; ^17^ Population Health Data Center National Cheng Kung University Tainan Taiwan

**Keywords:** catatonic, death, natural causes, suicide, unnatural causes

## Abstract

**Aim:**

To determine whether catatonia is associated with increased long‐term all‐cause and cause‐specific mortality.

**Methods:**

Using Taiwan's National Health Insurance Database (2000–2022), we assembled a population‐based cohort of all adults (≥18 years) with catatonia and matched each to four controls without catatonia on sex and birthdate. Mortality was compared between (1) individuals with catatonia and their unaffected siblings and (2) individuals with schizophrenia spectrum disorders with catatonia and those with schizophrenia spectrum disorders without catatonia. The primary outcome was all‐cause mortality; secondary outcomes were natural‐ and unnatural‐cause deaths. Adjusted hazard ratios (HRs) with 95% confidence intervals (CIs) were estimated with Cox models controlling for age, sex, socioeconomic status, urbanization level, and comorbidities.

**Results:**

We included 6642 individuals with catatonia and 26,568 matched controls. Over mean follow‐ups of 11.4 and 13.1 years, respectively, 2150 *versus* 3459 deaths occurred (adjusted HR 2.60, 95% CI 2.46–2.75). Risks were higher for natural causes (2.42, 2.28–2.57) and unnatural causes (5.57, 4.59–6.77). Compared with unaffected siblings, catatonia remained associated with excess all‐cause (1.82, 1.34–2.49), natural (1.57, 1.07–2.30), and unnatural mortality (2.73, 1.56–4.77). Within schizophrenia spectrum disorders, catatonia conferred higher all‐cause (1.20, 1.12–1.28) and natural mortality (1.27, 1.18–1.36), whereas unnatural mortality was similar (1.01, 0.87–1.17).

**Conclusions:**

Catatonia conferred a substantial, independent risk of premature mortality across multiple causes. Clinicians should recognize that catatonia is a serious disorder with long‐term consequences and should remain vigilant to prevent and manage complications beyond the acute episode.

Catatonia is a complex neuropsychiatric syndrome characterized by prominent psychomotor abnormalities—stuporous immobility, mutism, stereotyped movements, and rigidity.^1^, ^2^ Once considered a subtype of schizophrenia, it is now recognized that catatonic symptoms can accompany a wide range of conditions,^3^ including mood and neurodevelopmental disorders, general medical illnesses, and certain drug reactions.^4^ Epidemiologic surveys place its global incidence at roughly 5–10 per 100,000 person‐years and indicate that about 9% of psychiatric patients develop a catatonic syndrome,^5^, ^6^, ^7^ yet it often goes underdiagnosed in routine practice.^1^

Malignant catatonia—the most severe presentation—features autonomic instability (fever, labile blood pressure, tachycardia) and, if not treated promptly with benzodiazepines or electroconvulsive therapy, carries a fatality rate approaching 50%.^8^, ^9^ Even after successful treatment of an acute catatonic episode, patients may suffer long‐term adverse outcomes. For instance, survivors of catatonia face heightened risks of complications such as deep vein thrombosis, aspiration pneumonia, or malnutrition – all of which can cause lasting morbidity.^2^ These post‐catatonia complications could plausibly raise the risk of premature death. However, whether a history of catatonia actually translates into increased mortality risk remains unclear due to a lack of robust data. Most existing studies have been small (case reports or limited cohorts) with inconsistent findings.^10^, ^11^, ^12^ For example, one analysis noted 7% in‐hospital mortality among schizophrenia patients with catatonia *versus* 1.6% in those without.^10^ Conversely, another large inpatient cohort (approximately 800 catatonia cases) tracked 7 years post‐discharge and found equal adjusted mortality with contemporaneous psychiatric inpatients with other disorders.^11^ Given the paucity of definitive data and inconsistent results, well‐powered studies are needed to determine whether catatonia is associated with higher long‐term all‐cause or cause‐specific mortality.

In this context, we conducted a population‐based cohort study using 22 years of data in Taiwan to examine the association between catatonia and subsequent mortality. More than 6000 individuals with catatonia were each matched to a population control without catatonia to assess excess deaths. We also performed a sibling‐comparison analysis (comparing catatonic individuals to their unaffected full siblings) to control for shared genetic and early‐life environmental factors. Finally, we identified patients with catatonia who also had schizophrenia‐spectrum disorders and compared them with patients who had schizophrenia‐spectrum disorders but no catatonia, allowing us to determine whether any excess mortality is specific to catatonia rather than the baseline risk of serious mental illness.

## Methods

### Data Sources and Study Cohort

This cohort study adhered to the REporting of studies Conducted using Observational Routinely‐collected health Data (RECORD) guidelines (Appendix S1).^13^ The research was conducted ethically in accordance with the World Medical Association Declaration of Helsinki. The Institutional Review Board of Chang Gung Memorial Hospital approved the study protocol and waived the need for informed consent (No.: 202300262B0).

We utilized the Taiwan National Health Insurance Research Database (NHIRD), a nationwide claims repository launched in 1996 that captures medical information from Taiwan's National Health Insurance (NHI) system for more than 99% of Taiwan's residents. Because the NHI system offers low‐cost, easily accessible care, virtually all healthcare contacts are captured. Accordingly, non‐mortality attrition is negligible, with death being the predominant reason for follow‐up termination. Additionally, the NHIRD provides de‐identified, linkable records for every beneficiary. Available data include (1) demographic characteristics such as birth date, sex, income‐based insurance premium, residential region, and kinship ties between policyholders and dependents; (2) complete outpatient and inpatient encounter files with visit dates and International Classification of Diseases (ICD) codes; and (3) a death registry containing the exact date and certified cause of death. To preserve confidentiality, all personal identifiers are encrypted, and each individual is assigned a unique anonymous key that permits secure record linkage across datasets. For this analysis, we extracted NHIRD data from 2000 through 2022. Health conditions were identified with ICD‐9 codes for 2000–2015 and ICD‐10 codes for 2016–2022.^14^ The overall study workflow, including eligibility criteria and cohort construction, is illustrated in Figure S1.

We constructed the patient cohort as follows: (1) we first enrolled individuals who had received ≥2 clinical diagnoses of catatonia (ICD‐9: 295.2 and 293.89; ICD‐10: F20.2 and F06.1) from a board‐certified psychiatrist between January 1, 2001, and December 31, 2021; (2) we excluded patients with unspecified date of birth or sex to ensure complete demographic data; and (3) we restricted the analytic sample to adults aged ≥18 years at the initial catatonia diagnosis, thereby focusing on an adult population. To contextualize mortality risk, we formed three comparison cohorts. (1) Healthy‐matched control cohort: for each individual with catatonia, four individuals from the general NHIRD population who had no record of catatonia across their entire claims history were randomly selected and individually matched on identical sex and a birth‐date window of ±6 months. (2) Unaffected sibling cohort: among catatonic patients with at least one sibling identified in the database, we defined siblings as beneficiaries sharing ≥1 biological parent; the cohort comprised each index case together with all biologically related, unaffected brothers or sisters, furnishing a within‐family comparison. (3) Schizophrenia spectrum disorder without catatonia cohort: because psychosis‐related catatonia constituted the largest clinical subgroup in our sample, we also compared mortality between patients with schizophrenia spectrum disorders with catatonia against others with schizophrenia spectrum disorders without catatonia. Using criteria analogous to the catatonia cohort, we identified individuals who carried diagnoses of schizophrenia spectrum disorders (ICD‐9: 295, 297, 298.1, 298.3, 298.4, 298.8, and 298.9; ICD‐10: F20, F22‐F25, F28, and F29) but no recorded catatonic episode during the study period (2001–2021). Comparing this cohort with the catatonic schizophrenia spectrum disorder subgroup allowed us to assess whether excess mortality is attributable specifically to catatonia rather than to psychosis *per se*.

Participants in the three comparison cohorts were tracked prospectively from cohort‐specific index dates: (1) for the general population controls, follow‐up began on the calendar date that corresponded to the first confirmed catatonia diagnosis of the paired individual with catatonia; (2) for the unaffected sibling cohort, observation started on the earliest date for which comprehensive NHIRD coverage was available for each sibling; and (3) for the schizophrenia spectrum disorder without catatonia cohort, follow‐up commenced on the date of the first recorded psychotic disorder diagnosis. In every cohort, surveillance continued until the earliest occurrence of death or the end of the study period (December 31, 2022).

### Outcomes and Covariates

We ascertained mortality through linkage with the NHIRD Cause of Death Registry and followed participants from 2001 to 2022. The primary endpoint was all‐cause mortality. Secondary endpoints were cause‐specific deaths, grouped by ICD chapter into (1) natural causes—all deaths except those assigned to the “external causes of morbidity and mortality”; and (2) unnatural causes, comprising all external‐cause codes. Unnatural deaths were further subdivided into causes of accident, suicide, and assault/homicide. Table S1 lists the ICD codes corresponding to each cause‐of‐death category. Deaths that could not be classified or had missing ICD codes were classified as unknown cause.

Baseline characteristics collected for every participant comprised (1) age at cohort entry, (2) sex, (3) individual income level, (4) degree of urbanization of the residential area, and (5) medical comorbidities. Income was stratified into quartiles relative to the matched‐control population—highest (>75th percentile), upper‐middle (50th–75th), lower‐middle (25th–50th), and lowest (≤25th percentile)—thereby providing a representative benchmark for socioeconomic status across the database. Residential urbanization, an established proxy for health‐care accessibility in Taiwan, was classified on a four‐tier scale (levels 1–4, most to least urbanized) based on local population density, age structure, proportion of agricultural employment, prevalence of post‐secondary education, and availability of medical providers.^15^ Medical comorbidity burden was summarized by the Charlson Comorbidity Index (CCI), a validated measure that integrates 17 chronic conditions—including dementia, cerebrovascular and peripheral vascular disease, myocardial infarction, congestive heart failure, chronic pulmonary disease, diabetes (controlled and uncontrolled), renal disease, peptic ulcer disease, liver disease (severe and non‐severe), rheumatologic disorders, malignancies (localized and metastatic), paraplegia, and acquired immunodeficiency syndrome—to quantify overall morbidity and predict long‐term mortality.^16^

### Statistical Analyses

Baseline characteristics were summarized as means ± standard deviations for continuous variables and counts (percentages) for categorical variables. Kaplan–Meier curves were plotted to visualize cumulative survival, and Cox proportional‐hazards models were used to estimate hazard ratios (HRs) with 95% confidence intervals (CIs), employing time since cohort entry as the underlying time scale. Analyses were first run for all‐cause mortality and then repeated for each cause‐specific outcome. Model 1 provided crude, unadjusted HRs; Model 2 additionally adjusted for birth year, sex, income quartile, urbanization level, and CCI. Missing covariate values were assigned to an “unknown” category and included as nominal variables in the models.

We performed two sensitivity analyses, three prespecified subgroup analyses, and two alternative‐comparator analyses. The sensitivity analysis repeated the primary Cox models (model 1 and model 2) in (1) a complete‐case sample excluding participants with missing covariate data, and (2) a sample restricted to patients with ≥3 clinical diagnoses of catatonia from a board‐certified psychiatrist. Subgroup analyses stratified the models by age (adults 18–65 years *vs*. older adults ≥65 years), sex (male *vs*. female), and underlying etiology of catatonia (−psychosis‐related *vs*. nonpsychosis‐related). The comparator analyses contrasted (1) patients with catatonia who had ≥1 sibling with their unaffected siblings; and (2) patients with schizophrenia spectrum disorders with catatonia with those who had schizophrenia spectrum disorders without catatonia. The same statistical framework was applied, but model 2 in the sibling comparison additionally adjusted for sibling birth order and used cluster‐robust (sandwich) standard errors to account for within‐family correlation.^17^ All statistical procedures were executed in SAS 9.4 (SAS Institute Inc., Cary, NC, USA), with two‐sided *P* <0.05 considered statistically significant.

## Results

### Healthy‐Matched Control Analyses

Table 1 summarizes baseline characteristics for the 6642 adults with catatonia and 26,568 matched controls identified between 2001 and 2021. The mean (standard deviation) age at first catatonia diagnosis was 45.5 (18.0) years, and 56.4% were male. Average follow‐up spanned 11.4 years for the catatonia group and 13.1 years for controls. Throughout the study period, patients with catatonia were recorded in the NHIRD a mean (standard deviation) of 21 (38) times. Compared with controls, patients with catatonia were more likely to fall in the lower half of the income distribution (50th–100th percentile, 68.4% *vs*. 45.4%) and to have a higher comorbidity burden (CCI ≥3, 62.6% *vs*. 43.6%).

**Table 1:** Characteristics of all included participants from 2001 to 2021

	Matched healthy controls		Unaffected siblings		Schizophrenia spectrum disorders	
Characteristics	Case (*n* = 6642)	Control (*n* = 26,568)	Case (*n* = 1675)	Control (*n* = 2666)	Case (*n* = 4169)	Control (*n* = 306,585)
Basic information						
Age, year	45.5 ± 18.1	45.5 ± 18.1	–	–	40.5 ± 14.3	43.1 ± 16.8
Sex, male	3744 (56.4)	14,976 (56.4)	996 (59.5)	1340 (50.3)	2233 (53.6)	155,787 (50.8)
Personal income level						
>25th (highest)	429 (6.5)	6247 (23.5)	97 (5.8)	529 (19.8)	177 (4.2)	23,271 (7.6)
25–50th	1143 (17.2)	6418 (24.2)	310 (18.5)	760 (28.5)	632 (15.2)	57,527 (18.8)
50–75th	1296 (19.5)	6193 (23.3)	278 (16.6)	596 (22.4)	817 (19.6)	63,753 (20.8)
≤75th (lowest)	3245 (48.9)	5869 (22.1)	866 (51.7)	681 (25.5)	2270 (54.4)	141,553 (46.2)
Unknown	529 (8.0)	1841 (6.9)	124 (7.4)	100 (3.8)	273 (6.5)	20,481 (6.7)
Personal urbanization level						
Level 1 (urban)	3448 (51.9)	14,285 (53.8)	925 (55.2)	1517 (56.9)	2137 (51.3)	159,245 (51.9)
Level 2	2498 (37.6)	9429 (35.5)	606 (36.2)	947 (35.5)	1599 (38.4)	114,452 (37.3)
Level 3	495 (7.5)	1989 (7.5)	101 (6.0)	152 (5.7)	303 (7.3)	24,326 (7.9)
Level 4 (rural)	80 (1.2)	245 (0.9)	17 (1.0)	20 (0.8)	53 (1.3)	3869 (1.3)
Unknown	121 (1.8)	620 (2.3)	26 (1.6)	30 (1.1)	77 (1.8)	4693 (1.5)
Medical comorbidities						
0 (Charlson Comorbidity Index)	592 (8.9)	5889 (22.2)	272 (16.2)	747 (28)	472 (11.3)	40,756 (13.3)
1–2	1889 (28.4)	9087 (34.2)	734 (43.8)	1310 (49.1)	1441 (34.6)	98,803 (32.2)
≥3	4161 (62.6)	11,592 (43.6)	669 (39.9)	609 (22.8)	2256 (54.1)	167,026 (54.5)

^†^ Data was expressed as mean ± standard deviation or *N* (percentage).

**Fig. 1:**
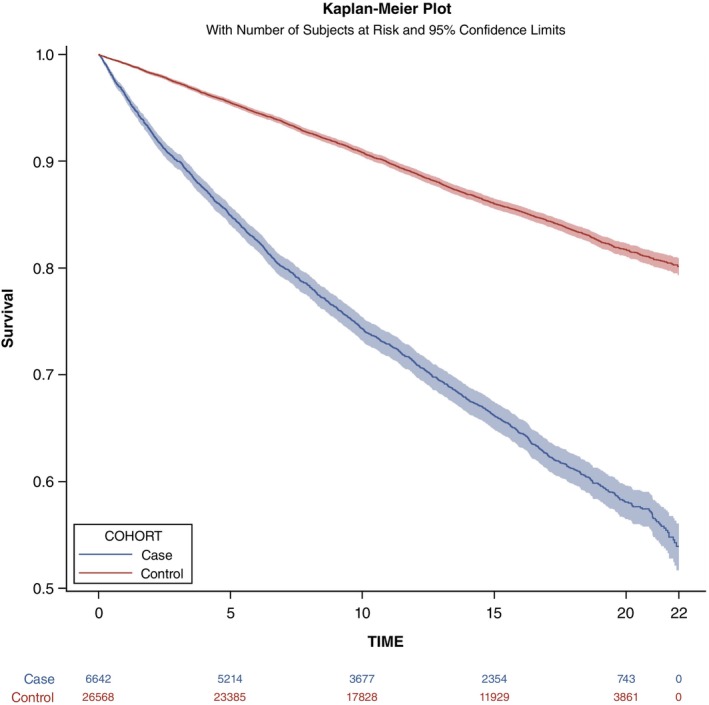
Kaplan–Meier survival curves for all‐cause mortality in patients with catatonia and matched healthy controls. Case: patients with catatonia; control: matched healthy controls.

During follow‐up, 2150 patients and 3459 matched controls died, corresponding to crude mortality rates of 2848 and 991 deaths per 10,000 person‐years, respectively (Tables 2 and S2). Kaplan–Meier curves show a pronounced separation beginning early and widening over time (Fig. 1). Both crude (model 1) and adjusted (model 2) HRs indicated a 2‐ to 3‐fold increase in all‐cause mortality among patients (model 1: HR 2.87, 95% CI 2.72–3.03; model 2: HR 2.60, 95% CI 2.46–2.75). Model 2 also showed elevated risks for natural (2.42, 2.28–2.57), unnatural (5.57, 4.59–6.77), and unknown causes of death (3.60, 1.90–6.80). Compared with unaffected participants, patients with catatonia had higher mortality risks across almost every natural‐cause category, including infectious and parasitic diseases; neoplasms; diseases of the blood and blood‐forming organs and certain disorders; endocrine, nutritional, and metabolic diseases; mental and behavioral disorders; diseases of the nervous system; diseases of the circulatory system; diseases of the respiratory system; diseases of the digestive system; diseases of the skin and subcutaneous tissue; diseases of the musculoskeletal system and connective tissue; diseases of the genitourinary system; and symptoms, signs and abnormal clinical and laboratory findings. They also faced increased risks of nearly all unnatural causes—accident and suicide.

**Table 2:** The risk of all‐cause and cause‐specific mortality among patients with catatonia *versus* healthy matched controls

Characteristics	Case, event (*n* = 6642)	Control, event (*n* = 26,568)	Adjusted hazard ratio (model 2)^‡^
All‐cause	2150 (32.4)	3459 (13.0)	2.60 (2.46–2.75)*
Natural causes	1856 (27.9)	3214 (12.1)	2.42 (2.28–2.57)*
Certain infectious and parasitic diseases	80 (1.2)	106 (0.4)	2.87 (2.12–3.87)*
Neoplasms	329 (5.0)	951 (3.6)	1.36 (1.19–1.55)*
Diseases of the blood and blood‐forming organs and certain disorders	7 (0.1)	10 (<0.1)	3.18 (1.14–8.87)*
Endocrine, nutritional, and metabolic diseases	163 (2.5)	223 (0.8)	3.02 (2.45–3.73)*
Mental and behavioral disorders	76 (1.1)	51 (0.2)	6.96 (4.75–10.20)*
Diseases of the nervous system	72 (1.1)	49 (0.2)	6.81 (4.63–10.03)*
Diseases of the eye and adnexa	0 (0.0)	0 (0.0)	–
Diseases of the ear and mastoid process	0 (0.0)	0 (0.0)	–
Diseases of the circulatory system	417 (6.3)	828 (3.1)	2.26 (2.00–2.55)*
Diseases of the respiratory system	306 (4.6)	421 (1.6)	3.08 (2.65–3.58)*
Diseases of the digestive system	159 (2.4)	196 (0.7)	3.00 (2.41–3.73)*
Diseases of the skin and subcutaneous tissue	12 (0.2)	20 (0.1)	2.67 (1.28–5.59)*
Diseases of the musculoskeletal system and connective tissue	18 (0.3)	29 (0.1)	2.33 (1.26–4.29)*
Diseases of the genitourinary system	108 (1.6)	166 (0.6)	2.72 (2.12–3.49)*
Pregnancy, childbirth, and the puerperium	0 (0.0)	1 (<0.1)	–
Certain conditions originating in the perinatal period	0 (0.0)	0 (0.0)	–
Congenital malformations, deformations and chromosomal abnormalities	2 (<0.1)	2 (<0.1)	4.09 (0.51–32.87)
Symptoms, signs and abnormal clinical and laboratory findings, not elsewhere classified	107 (1.6)	161 (0.6)	3.11 (2.40–4.04)*
Unnatural causes	276 (4.2)	218 (0.8)	5.57 (4.59–6.77)*
Accident	140 (2.1)	127 (0.5)	4.99 (3.83–6.49)*
Suicide	134 (2.0)	86 (0.3)	6.82 (5.08–9.16)*
Assault/Homicide	2 (<0.1)	5 (<0.1)	0.93 (0.17–5.25)
Unknown causes	18 (0.3)	27 (0.1)	3.60 (1.90–6.80)*

^†^ Event was expressed as N (percentage).

^‡^ Model 2 adjusted for all variables (birth year, sex, income level, urbanization level, and Charlson Comorbidity Index).

^§^ Asterisks indicate statistical significance.

The results were consistent across sensitivity analyses limited to the complete‐case sample and to patients with ≥3 clinical diagnoses of catatonia, as well as across stratified analyses by age, sex, and etiology. In model 2, patients with catatonia had an increased risk of all‐cause deaths (HR, 2‐fold to 3‐fold), natural deaths (HR, 2‐fold to 3‐fold), unnatural deaths (HR, 3‐fold to 6‐fold), and unknown‐cause deaths (HR, 3‐fold to 5‐fold) (Tables S3–S10).

### Unaffected Sibling Analysis

We identified 1675 patients with catatonia and 2666 unaffected siblings. Compared with their siblings, patients were more often male (59.5% *vs*. 50.3%), fell more frequently into the lower half of the income distribution (50th–100th percentile: 68.3% *vs*. 47.9%), and had a higher comorbidity burden (CCI ≥3: 39.9% *vs*. 22.8%) (Table 1). During follow‐up, 161 patients and 89 siblings died, corresponding to crude mortality rates of 451 and 154 deaths per 10,000 person‐years, respectively. In model 2, catatonia was associated with elevated risks for all‐cause mortality (HR 1.82, 95% CI 1.34–2.49), natural‐cause mortality (1.57, 1.07–2.30), and unnatural‐cause mortality (2.73, 1.56–4.77). These risks paralleled those observed in the primary matched‐control analysis, although the absolute values were slightly attenuated. Estimates for individual causes of death were less precise because of the small number of events and limited statistical power (Tables 3 and S11).

**Table 3:** The risk of all‐cause and cause‐specific mortality among patients with catatonia *versus* their unaffected siblings

Characteristics	Case, event (*n* = 1675)	Control, event (*n* = 2666)	Adjusted hazard ratio (model 2)
All‐cause	161 (9.6)	89 (3.3)	1.82 (1.34–2.49)*
Natural causes	111 (6.6)	62 (2.3)	1.57 (1.07–2.30)*
Certain infectious and parasitic diseases	5 (0.3)	1 (<0.1)	4.63 (0.32–67.90)
Neoplasms	19 (1.1)	12 (0.5)	1.64 (0.70–3.82)
Diseases of the blood and blood‐forming organs and certain disorders	0 (0.0)	0 (0.0)	–
Endocrine, nutritional, and metabolic diseases	5 (0.3)	4 (0.2)	0.57 (0.16–2.03)
Mental and behavioral disorders	12 (0.7)	1 (<0.1)	10.40 (1.25–86.87)*
Diseases of the nervous system	7 (0.4)	7 (0.3)	0.69 (0.13–3.55)
Diseases of the eye and adnexa	0 (0.0)	0 (0.0)	–
Diseases of the ear and mastoid process	0 (0.0)	0 (0.0)	–
Diseases of the circulatory system	24 (1.4)	13 (0.5)	2.09 (0.95–4.59)
Diseases of the respiratory system	16 (1.0)	6 (0.2)	2.66 (0.78–9.05)
Diseases of the digestive system	11 (0.7)	6 (0.2)	1.40 (0.44–4.46)
Diseases of the skin and subcutaneous tissue	1 (0.1)	1 (<0.1)	3.07 (0.42–22.58)
Diseases of the musculoskeletal system and connective tissue	3 (0.2)	1 (<0.1)	2.02 (0.23–17.42)
Diseases of the genitourinary system	1 (0.1)	2 (0.1)	0.10 (0.00–7.81)
Pregnancy, childbirth, and the puerperium	0 (0.0)	0 (0.0)	–
Certain conditions originating in the perinatal period	0 (0.0)	0 (0.0)	–
Congenital malformations, deformations and chromosomal abnormalities	0 (0.0)	1 (<0.1)	–
Symptoms, signs and abnormal clinical and laboratory findings, not elsewhere classified	7 (0.4)	7 (0.3)	0.86 (0.32–2.35)
Unnatural causes	48 (2.9)	26 (1.0)	2.73 (1.56–4.77)*
Accident	23 (1.4)	11 (0.4)	2.95 (1.34–6.49)*
Suicide	24 (1.4)	15 (0.6)	2.51 (1.13–5.58)*
Assault/Homicide	1 (0.1)	0 (0.0)	–
Unknown causes	2 (0.1)	1 (<0.1)	2.01 (0.06–72.36)

^†^ Event was expressed as N (percentage).

^‡^ Model 2 adjusted for all variables (birth year, sex, income level, urbanization level, Charlson Comorbidity Index, and sibling birth order).

^§^ Asterisks indicate statistical significance.

### Schizophrenia Spectrum Disorder Analysis

We identified 4169 patients with catatonia and schizophrenia spectrum disorders and 306,585 patients with schizophrenia spectrum disorders without catatonia. Compared with those without catatonia, the catatonia group was younger (40.5 years *vs*. 43.1 years), more often male (53.6% *vs*. 50.8%), and more likely to fall within the lower half of the income distribution (50th–100th percentile: 74.0% *vs*. 67.0%) (Table 1). During follow‐up, 954 patients with catatonia and 76,241 patients without catatonia died, corresponding to crude mortality rates of 184.5 and 197.3 deaths per 10,000 person‐years, respectively. In model 2, the risks of all‐cause mortality (HR 1.20, 95% CI 1.12–1.28) and natural‐cause mortality (1.27, 1.18–1.36) were similar to those from the primary matched‐control and sibling analyses, though the risk values were further attenuated (Tables 4 and S12). When we examined individual natural‐cause categories, the direction of effect remained the same—higher risk in the catatonia group—but statistical significance emerged only for some categories (certain infectious and parasitic diseases; neoplasms; endocrine, nutritional, and metabolic diseases; mental and behavioral disorders; diseases of the respiratory system). Notably, the overall risk of death from unnatural causes did not differ between groups (1.01, 0.87–1.17). However, within the unnatural‐cause category, patients with catatonia remained at elevated hazard for accident (1.33, 1.07–1.67), but not for suicide or assault/homicide.

**Table 4:** The risk of all‐cause and cause‐specific mortality among patients with schizophrenia spectrum disorders with *versus* without catatonia

Characteristics	Case, event (n = 4169)	Control, event (n = 306,585)	Adjusted hazard ratio (model 2)
All‐cause	954 (22.9)	76,241 (24.9)	1.20 (1.12–1.28)*
Natural causes	771 (18.5)	62,616 (20.4)	1.27 (1.18–1.36)*
Certain infectious and parasitic diseases	36 (0.9)	2194 (0.7)	1.70 (1.22–2.36)*
Neoplasms	138 (3.3)	10,814 (3.5)	1.23 (1.04–1.46)*
Diseases of the blood and blood‐forming organs and certain disorders	4 (0.1)	218 (0.1)	1.87 (0.69–5.03)
Endocrine, nutritional, and metabolic diseases	75 (1.8)	5628 (1.8)	1.32 (1.05–1.66)*
Mental and behavioral disorders	46 (1.1)	2764 (0.9)	1.74 (1.30–2.32)*
Diseases of the nervous system	28 (0.7)	1849 (0.6)	1.44 (0.99–2.09)
Diseases of the eye and adnexa	0 (0.0)	1 (<0.1)	–
Diseases of the ear and mastoid process	0 (0.0)	3 (<0.1)	–
Diseases of the circulatory system	167 (4.0)	15,490 (5.1)	1.14 (0.97–1.32)
Diseases of the respiratory system	116 (2.8)	10,299 (3.4)	1.35 (1.13–1.62)*
Diseases of the digestive system	67 (1.6)	4987 (1.6)	1.15 (0.90–1.46)
Diseases of the skin and subcutaneous tissue	6 (0.1)	346 (0.1)	1.86 (0.83–4.18)
Diseases of the musculoskeletal system and connective tissue	6 (0.1)	507 (0.2)	1.18 (0.53–2.65)
Diseases of the genitourinary system	32 (0.8)	3359 (1.1)	1.12 (0.79–1.58)
Pregnancy, childbirth, and the puerperium	0 (0.0)	4 (<0.1)	–
Certain conditions originating in the perinatal period	0 (0.0)	0 (0.0)	–
Congenital malformations, deformations and chromosomal abnormalities	2 (<0.1)	53 (<0.1)	2.45 (0.60–10.09)
Symptoms, signs and abnormal clinical and laboratory findings, not elsewhere classified	48 (1.2)	4100 (1.3)	1.17 (0.88–1.55)
Unnatural causes	178 (4.3)	13,020 (4.2)	1.01 (0.87–1.17)
Accident	79 (1.9)	4606 (1.5)	1.33 (1.07–1.67)*
Suicide	98 (2.4)	8287 (2.7)	0.85 (0.70–1.04)
Assault/Homicide	1 (<0.1)	127 (<0.1)	0.54 (0.08–3.89)
Unknown causes	5 (0.1)	605 (0.2)	0.99 (0.41–2.38)

^†^ Event was expressed as *N* (percentage).

^‡^ Model 2 adjusted for all variables (birth year, sex, income level, urbanization level, and Charlson Comorbidity Index).

^§^ Asterisks indicate statistical significance.

## Discussion

In this population‐based cohort, individuals with catatonia had a 2.6‐fold higher risk of all‐cause mortality than healthy‐matched controls. Excess mortality extended to both natural causes (2.4‐fold increase) and unnatural causes (5.6‐fold increase). These elevations persisted across all age groups (adults and older adults), both sexes, and etiologic subtypes (psychotic‐related and non‐psychotic‐related). Relative to unaffected siblings, the associations remained but were modestly attenuated (all‐cause, 1.8‐fold; natural, 1.6‐fold; unnatural, 2.7‐fold). When the comparison was confined to schizophrenia spectrum disorders, patients whose psychosis was accompanied by catatonia retained higher all‐cause (1.2‐fold) and natural‐cause (1.3‐fold) mortality than those with psychosis without catatonia, whereas the excess risk for unnatural deaths was no longer observed.

The demographic characteristics of our Taiwanese catatonia cohort are broadly consistent with recent population‐based studies from the United Kingdom and the United States.^7^, ^11^, ^18^ Rogers *et al*. reported a mean age of 37 years and male predominance (54%) in their London cohort of 787 catatonia patients,^11^ and Luccarelli *et al*. reported a mean age of 48 years and a male proportion of 45% in their United States cohort of 16,575 catatonia patients.^18^ In comparison, our cohort showed a mean age of 46 years and 56% male composition. The similarity in demographic profiles across three geographically and culturally distinct populations (Taiwan, the United Kingdom, and the United States) suggests that catatonia may affect similar demographic groups across different healthcare systems and ethnic backgrounds, thereby supporting the potential comparability of our mortality findings. However, prior research on long‐term all‐cause mortality in catatonia is extremely limited and fragmented. To our knowledge, only two smaller studies are available. A Japanese inpatient study that compared schizophrenia with catatonia and schizophrenia without catatonia reported significantly higher mortality among catatonic patients,^10^ in line with our findings (Table 4)‐. In contrast, a UK inpatient cohort found no mortality difference between patients with catatonia and other inpatients with psychiatric disorders without catatonia during 7 years of postdischarge followup.^11^ This discrepancy may stem from its inpatient‐only sample, the broad mental‐disorder control group, and the lack of adjustment for medical comorbidities—factors that can mask true risk. By offering three complementary comparisons in our study—catatonia *versus* healthy‐matched controls, catatonia *versus* unaffected siblings, and catatonic psychotic disorders *versus* non‐catatonic psychotic disorders—our study provides the most comprehensive evidence to date and consistently demonstrates excess all‐cause mortality among individuals with catatonia.

In our cause‐specific analyses, catatonia remained strongly linked to excess mortality from both natural and unnatural causes, regardless of whether healthy‐matched controls or unaffected siblings were used as the reference group. For natural deaths, virtually every ICD chapter showed elevated hazards in the catatonia cohort—including infections; neoplasms; hematologic, endocrine, nutritional, and metabolic disorders; mental disorders; and diseases of the nervous, circulatory, respiratory, digestive, dermatologic, musculoskeletal, and genitourinary systems. Although the sibling comparison suffered from reduced statistical power owing to the small number of events, it reproduced similar broad pattern of excess risk (Table 3). These findings are biologically plausible: prolonged catatonic immobility promotes dehydration,^19^ malnutrition,^20^ deep vein thrombosis with potentially fatal pulmonary embolism,^21^, ^22^ recurrent aspiration pneumonia, urinary tract infection, or pressure ulcer sepsis^23^, ^24^—complications that often go unnoticed when patients are mute and inert for weeks to months, ultimately driving long‐term natural‐cause mortality. For unnatural deaths, both the primary and siblings' analytic frameworks identified accidents (adjusted HRs 5.1 and 3.4) and suicides (7.0 and 2.6) as the dominant contributors (Tables 2 and 3). Accidental deaths likely reflect abrupt transitions from frozen stupor to violent, purposeless agitation,^25^, ^26^ leaving patients oblivious to danger and vulnerable to falls, choking, and other traumatic injuries. Elevated suicide risk probably stems from severe psychotic symptoms coupled with profound hopelessness, or comorbid mood symptoms or disorders.^5^, ^27^

Across strata defined by age (adults and older adults), sex (male and female), and underlying etiology (psychosis‐related and non‐psychosis‐related catatonia), catatonia was linked to 2–3‐fold higher all‐cause and natural‐cause mortality and 3–6‐fold higher unnatural‐cause mortality. The consistency of these elevations indicates that the excess risk is largely independent of demographic or diagnostic context and supports the Diagnostic and Statistical Manual of Mental Disorders‐Fifth Edition decision to classify catatonia separately from primary psychotic disorders. In comparisons restricted to schizophrenia spectrum disorders, catatonia retained higher hazards for all‐cause and natural‐cause deaths after multivariable adjustment, although several natural cause‐specific estimates lost statistical significance (Table 4). This likely reflects multiple factors. First, both groups (with and without catatonia) share a high baseline mortality risk from schizophrenia, which narrows the mortality gap attributable specifically to catatonia. Second, the smaller number of deaths in certain cause‐specific categories reduced statistical power to detect differences. Third, shared risk factors common to all patients with schizophrenia spectrum disorders, such as long‐term antipsychotic medication use and its effects on circulatory system diseases, may have partially masked the independent contribution of catatonia.

Several limitations merit consideration. First, the NHIRD lacks catatonia‐specific rating scales, episode frequency data, detailed etiologic subtypes (drug‐induced or disease‐induced), and lifestyle covariates (diet, smoking, occupational exposures). This likely biased the estimates toward the null, as unmeasured confounders contribute to mortality in both groups. Second, the NHIRD lacks detailed treatment information, including treatment response to specific interventions (benzodiazepines and electroconvulsive therapy) and duration of catatonic episodes. This prevents the examination of differences in treatment effectiveness between survivors and decedents. The direction of bias is uncertain and could be bidirectional. If the catatonia group received more effective treatment (due to heightened clinical attention to severe cases), our estimates might underestimate the intrinsic mortality risk of catatonia. Conversely, if the catatonia group faced treatment barriers or delays, our estimates might have overestimated mortality risk by capturing inadequately treated cases. Third, catatonia diagnoses in the database are psychiatrist‐assigned rather than assessment‐tool‐based, and requiring ≥2 psychiatrist‐assigned ICD codes was used to enhance diagnostic accuracy and reduce misclassification. This criterion likely enriched the cohort for “manifest” catatonia and may have excluded milder or subclinical presentations, or patients who died early. The direction of bias is uncertain: if excluded cases had lower mortality (mild cases not requiring follow‐up), our estimates would overestimate risk; if excluded cases had higher mortality (severe cases dying before re‐evaluation), our estimates would underestimate risk. Fourth, individuals younger than 18 years were not studied,^28^ limiting the applicability of our conclusions to adolescents. This exclusion does not directly bias our adult mortality estimates but limits generalizability if catatonia‐related mortality risks differ substantially by age group. Fifth, this study did not compare catatonia‐associated mortality risk in those with other specific psychiatric diagnoses (e.g., affective disorders, neurodevelopmental disorders). This does not directly bias our estimates but limits our ability to determine whether the observed mortality risk associated with catatonia in schizophrenia spectrum disorders is also common across a wider range of serious mental illnesses. This underscores the need for future studies to compare mortality outcomes across a broader range of psychiatric disorder groups. Finally, conducting the study solely in Taiwan limits generalizability to other healthcare systems. The direction of bias for Taiwanese population estimates is minimal, but applicability to other populations is uncertain given cross‐country variation in healthcare quality and practices. This underscores the need for replication in other jurisdictions.

## Conclusions

In this population‐based matched, sibling, and psychotic‐cohort study, catatonia conferred broadly elevated mortality. *Versus* matched healthy controls, risks were higher for all‐cause, natural, and unnatural deaths across ages, sexes, and etiologies (psychosis/non‐psychosis). Although attenuated, these excesses remained significant when patients with catatonia were compared with unaffected siblings. Within schizophrenia spectrum disorders, patients with catatonia still showed greater all‐cause and natural‐cause mortality than patients without catatonia; overall unnatural mortality was neutral, but accident‐related deaths were more frequent, whereas suicide risk was similar. These findings highlight catatonia's distinctive and independent impact on long‐term survival, and signal the need for clinicians to extend vigilance beyond acute management. Future research should delineate underlying mechanisms, identify prognostic predictors for post‐episode mortality, and inform surveillance, preventive, and early‐intervention strategies.

## Funding Information

This study is supported by grants from the Taiwan National Science and Technology Council (112‐2314‐B‐182‐070‐MY3). The funder had no role in the design and conduct of the study; collection, management, analysis, and interpretation of the data; preparation, review, or approval of the manuscript; and decision to submit the manuscript for publication. CWH is supported by Taiwan National Science and Technology Council (109‐2314‐B‐182A‐009‐MY2, 111‐2314‐B‐182A‐027‐, and 112‐2314‐B‐182‐070‐MY3) and the Chang Gung Medical Foundation (CMRPG8N0881, CMRPG8P0631, CORPG8P0561, and BMRPJ30).

## Disclosure statement

Marco Solmi has received honoraria/has been a consultant for AbbVie, Angelini, Bausch Health, Boehringer Ingelheim, Lundbeck, Otsuka, Teva. The other authors declare no financial interests or potential conflicts of interest regarding the authorship and publication of this article.

## Author contributions

Research idea and study design: CWH; data acquisition and interpretation: CAH and CWH; statistical analysis: CWH; manuscript drafting: CWH; manuscript revision: YCC, MS, CSL, MHC, YHY, LJW, and CCL. Each author contributed important intellectual content during manuscript drafting or revision and accepts accountability for the overall work by ensuring that questions pertaining to the accuracy or integrity of any portion of the work are appropriately investigated and resolved.

## Supporting information


**Data S1.** Supplementary Information.

Appendix S1.

## Data Availability

The data of the current study would be available to the corresponding author upon reasonable request and approval from the Institutional Review Board and permission from the Taiwan Ministry of Health and Welfare. Chih‐Wei Hsu had full access to all the data in the study and takes responsibility for the integrity of the data and the accuracy of the data analysis.
