# Supplementary material for: Catatonia and elevated mortality: A population‐wide cohort study with healthy, sibling, and schizophrenia spectrum controls
Source: Psychiatry Clin Neurosci. 2025 Nov 13;80(2):121–8. doi: 10.1111/pcn.13915 (PMC12866368; doi:10.1111/pcn.13915)
Supplement: Supplementary file 1 — Data S1. Supplementary Information. [file PCN-80-121-s002.docx]

**Supplementary material**

**Catatonia and Elevated Mortality: A Population-Wide Cohort Study with Healthy, Sibling, and** **Schizophrenia Spectrum Controls**

Chih-Wei Hsu, MD^a^, Yang-Chieh Brian Chen, MD^b*^, Marco Solmi, MD^c,d,e,f,g^, Chih-Sung Liang, MD^h,i^, Mu-Hong Chen, MD^j,k^, Yao-Hsu Yang, MD^l,m,n^, Liang-Jen Wang, MD^o*^, Edward Chia-Cheng Lai, PhD^p,q^

^a^ Department of Psychiatry, Kaohsiung Chang Gung Memorial Hospital and Chang Gung University College of Medicine, Kaohsiung, Taiwan

^b^ Department of Psychiatry and Behavioral Sciences, The University of Texas Health Science Center at Houston, Houston, TX, USA

^c^ Department of Child and Adolescent Psychiatry, Charité Universitätsmedizin, Berlin, Germany

^d^ Department of Psychiatry, University of Ottawa, Ottawa, Canada

^e^ Department of Mental Health, The Ottawa Hospital, Ottawa, Canada

^f^ Ottawa Hospital Research Institute, Ottawa, Canada

^g^ School of Epidemiology and Public Health, Faculty of Medicine, University of Ottawa, Ottawa, Canada

^h^ Department of Psychiatry, Beitou branch, Tri-Service General Hospital, National Defense Medical University, Taipei, Taiwan

^i^ Department of Psychiatry, National Defense Medical University, Taipei, Taiwan

^j^ Department of Psychiatry, Taipei Veterans General Hospital, Taipei, Taiwan

^k^ Department of Psychiatry, College of Medicine, National Yang Ming Chiao Tung University, Taipei, Taiwan

^l^ Department of Traditional Chinese Medicine, Chiayi Chang Gung Memorial Hospital, Chiayi, Taiwan

^m^ Health Information and Epidemiology Laboratory of Chang Gung Memorial Hospital, Chiayi, Taiwan

^n^ School of Traditional Chinese Medicine, College of Medicine, Chang Gung University, Taoyuan, Taiwan

^o^ Department of Child and Adolescent Psychiatry, Kaohsiung Chang Gung Memorial Hospital, Chang Gung University College of Medicine, Kaohsiung, Taiwan

^p^ School of Pharmacy, Institute of Clinical Pharmacy and Pharmaceutical Sciences, College of Medicine, National Cheng Kung University, Tainan, Taiwan

^q^ Population Health Data Center, National Cheng Kung University, Tainan, Taiwan

^*^**Contributed equally as corresponding authors**

| **Content** | **Page** |
| --- | --- |
| **eTable 1.** Diagnosis codes for causes of death | 1 |
| **eTable 2.** The risk of all-cause and cause-specific mortality among patients with catatonia versus healthy‑matched controls | 2 |
| **eTable 3.** The risk of all-cause and cause-specific mortality among patients with catatonia versus healthy‑matched controls, excluding participants with missing data | 3 |
| **eTable 4.** The risk of all-cause and cause-specific mortality among patients with catatonia versus healthy‑matched controls, with ≥3 clinical diagnoses of catatonia | 4 |
| **eTable 5.** The risk of all-cause and cause-specific mortality among patients with catatonia versus healthy‑matched controls, by age (adults) | 5 |
| **eTable 6.** The risk of all-cause and cause-specific mortality among patients with catatonia versus healthy‑matched controls, by age (older adults) | 6 |
| **eTable 7.** The risk of all-cause and cause-specific mortality among patients with catatonia versus healthy‑matched controls, by sex (male) | 7 |
| **eTable 8.** The risk of all-cause and cause-specific mortality among patients with catatonia versus healthy‑matched controls, by sex (female) | 8 |
| **eTable 9.** The risk of all-cause and cause-specific mortality among patients with catatonia versus healthy‑matched controls, by etiology (psychosis‑related) | 9 |
| **eTable 10.** The risk of all-cause and cause-specific mortality among patients with catatonia versus healthy‑matched controls, by etiology (non‑psychosis‑related) | 10 |
| **eTable 11.** The risk of all-cause and cause-specific mortality among patients with catatonia versus their unaffected siblings | 11 |
| **eTable 12.** The risk of all-cause and cause-specific mortality among patients with schizophrenia spectrum disorders with versus without catatonia | 12 |
| **eFigure 1.** Flowchart of the selection process for the study | 13 |

**eTable 1.** Diagnosis codes for causes of death

| Specific cause of death | ICD-9 codes | ICD-10 codes |
| --- | --- | --- |
| Natural causes | 001−799 | A−R |
| Certain infectious and parasitic diseases | 001−139 | A00−B99 |
| Neoplasms | 140−239 | C00−D49 |
| Diseases of the blood and blood-forming organs and certain disorders (involving the immune mechanism) | 279−289 | D50−D89 |
| Endocrine, nutritional, and metabolic diseases | 240−278 | E00−E90 |
| Mental and behavioral disorders | 290−319 | F00−F99 |
| Diseases of the nervous system | 320−359 | G00−G99 |
| Diseases of the eye and adnexa | 360−379 | H00−H59 |
| Diseases of the ear and mastoid process | 380−389 | H60−H95 |
| Diseases of the circulatory system | 390−459 | I00−I99 |
| Diseases of the respiratory system | 460−519 | J00−J99 |
| Diseases of the digestive system | 520−579 | K00−K93 |
| Diseases of the skin and subcutaneous tissue | 680−709 | L00−L99 |
| Diseases of the musculoskeletal system and connective tissue | 710−739 | M00−M99 |
| Diseases of the genitourinary system | 580−629 | N00−N99 |
| Pregnancy, childbirth, and the puerperium | 630−679 | O00−O99 |
| Certain conditions originating in the perinatal period | 760−779 | P00−P96 |
| Congenital malformations, deformations and chromosomal abnormalities | 740−759 | Q00−Q99 |
| Symptoms, signs and abnormal clinical and laboratory findings, not elsewhere classified | 780−799 | R00−R99 |
| Unnatural causes (external causes of morbidity and mortality) | E800−E969, E980−E989 | V01−Y98 |
| Accident | E800−E949 | V01−X59, Y40−Y86, Y88, Y89 |
| Suicide | E950−E959, E980−E989 | X60−X84, Y10−Y34 |
| Assault or Homicide | E960–E969 | X85−X99, Y00−Y09 |
| Unknown causes | No record or code other than the above | No record or code other than the above |

Abbreviation: ICD, International Classification of Diseases

**eTable 2.** The risk of all-cause and cause-specific mortality among patients with catatonia versus healthy‑matched controls

| Characteristics | Crude hazard ratio (model 1) |
| --- | --- |
| All-cause | 2.87 (2.72–3.03)* |
| Natural causes | 2.67 (2.52–2.82)* |
| Certain infectious and parasitic diseases | 3.48 (2.60–4.65)* |
| Neoplasms | 1.60 (1.41–1.82)* |
| Diseases of the blood and blood-forming organs and certain disorders | 3.13 (1.19–8.23)* |
| Endocrine, nutritional, and metabolic diseases | 3.36 (2.75–4.12)* |
| Mental and behavioral disorders | 6.95 (4.87–9.92)* |
| Diseases of the nervous system | 6.80 (4.73–9.78)* |
| Diseases of the eye and adnexa | – |
| Diseases of the ear and mastoid process | – |
| Diseases of the circulatory system | 2.34 (2.08–2.63)* |
| Diseases of the respiratory system | 3.34 (2.88–3.87)* |
| Diseases of the digestive system | 3.70 (3.00–4.56)* |
| Diseases of the skin and subcutaneous tissue | 2.77 (1.35–5.67)* |
| Diseases of the musculoskeletal system and connective tissue | 2.84 (1.57–5.11)* |
| Diseases of the genitourinary system | 2.99 (2.35–3.81)* |
| Pregnancy, childbirth, and the puerperium | – |
| Certain conditions originating in the perinatal period | – |
| Congenital malformations, deformations and chromosomal abnormalities | 4.81 (0.68–34.20) |
| Symptoms, signs and abnormal clinical and laboratory findings, not elsewhere classified | 3.08 (2.41–3.93)* |
| Unnatural causes | 5.79 (4.84–6.91)* |
| Accident | 5.09 (4.01–6.48)* |
| Suicide | 7.04 (5.37–9.23)* |
| Assault / Homicide | 1.72 (0.33-8.89) |
| Unknown causes | 3.27 (1.80–5.94)* |

^1^ Asterisks indicate statistical significance.

**eTable 3.** The risk of all-cause and cause-specific mortality among patients with catatonia versus healthy‑matched controls, excluding participants with missing data

| Characteristics | Case, event (n = 6005) | Control, event (n = 22,236) | Crude hazard ratio (model 1) | Adjusted hazard ratio (model 2) |
| --- | --- | --- | --- | --- |
| All-cause | 1829 (30.5) | 2346 (10.6) | 3.32 (3.13–3.53)* | 2.78 (2.60–2.96)* |
| Natural causes | 1568 (26.1) | 2152 (9.7) | 3.11 (2.91–3.32)* | 2.57 (2.40–2.75)* |
| Certain infectious and parasitic diseases | 66 (1.1) | 69 (0.3) | 4.06 (2.90–5.69)* | 2.97 (2.09–4.23)* |
| Neoplasms | 281 (4.7) | 685 (3.1) | 1.76 (1.53–2.02)* | 1.38 (1.19–1.59)* |
| Diseases of the blood and blood-forming organs and certain disorders | 6 (0.1) | 6 (<0.1) | 4.19 (1.35–13.00)* | 3.66 (1.10–12.15)* |
| Endocrine, nutritional, and metabolic diseases | 137 (2.3) | 133 (0.6) | 4.36 (3.43–5.53)* | 3.53 (2.75–4.52)* |
| Mental and behavioral disorders | 67 (1.1) | 35 (0.2) | 8.21 (5.45–12.36)* | 7.60 (4.90–11.81)* |
| Diseases of the nervous system | 63 (1.0) | 31 (0.1) | 8.68 (5.65–13.35)* | 8.14 (5.16–12.84)* |
| Diseases of the eye and adnexa | 0 (0.0) | 0 (0.0) | – | – |
| Diseases of the ear and mastoid process | 0 (0.0) | 0 (0.0) | – | – |
| Diseases of the circulatory system | 345 (5.7) | 533 (2.4) | 2.78 (2.43–3.18)* | 2.47 (2.14–2.84)* |
| Diseases of the respiratory system | 260 (4.3) | 263 (1.2) | 4.19 (3.53–4.98)* | 3.55 (2.98–4.24)* |
| Diseases of the digestive system | 142 (2.4) | 140 (0.6) | 4.27 (3.38–5.39)* | 3.23 (2.52–4.12)* |
| Diseases of the skin and subcutaneous tissue | 12 (0.2) | 13 (0.1) | 3.94 (1.80–8.65)* | 3.27 (1.45–7.36)* |
| Diseases of the musculoskeletal system and connective tissue | 14 (0.2) | 20 (0.1) | 2.96 (1.49–5.86)* | 2.14 (1.04–4.39)* |
| Diseases of the genitourinary system | 82 (1.4) | 116 (0.5) | 3.00 (2.26–3.98)* | 2.35 (1.76–3.14)* |
| Pregnancy, childbirth, and the puerperium | 0 (0.0) | 1 (<0.1) | – | – |
| Certain conditions originating in the perinatal period | 0 (0.0) | 0 (0.0) | – | – |
| Congenital malformations, deformations and chromosomal abnormalities | 1 (<0.1) | 1 (<0.1) | 4.45 (0.28–71.24) | 2.50 (0.12–53.82) |
| Symptoms, signs and abnormal clinical and laboratory findings, not elsewhere classified | 92 (1.5) | 106 (0.5) | 3.71 (2.80–4.90)* | 3.46 (2.56–4.67)* |
| Unnatural causes | 246 (4.1) | 177 (0.8) | 5.86 (4.83–7.11)* | 5.70 (4.62–7.04)* |
| Accident | 121 (2.0) | 101 (0.5) | 5.11 (3.92–6.65)* | 5.13 (3.84–6.85)* |
| Suicide | 124 (2.1) | 71 (0.3) | 7.28 (5.44–9.75)* | 7.07 (5.15–9.70)* |
| Assault / Homicide | 1 (<0.1) | 5 (<0.1) | 0.79 (0.09–6.80) | 0.42 (0.05–3.92) |
| Unknown causes | 15 (0.2) | 17 (0.1) | 3.99 (1.99–8.00)* | 4.15 (1.96–8.80)* |

^1^ Event was expressed as N (percentage).

^2^ Model 2 adjusted for all variables (birth year, sex, income level, urbanization level, and Charlson Comorbidity Index).

^3^ Asterisks indicate statistical significance.

**eTable 4.** The risk of all-cause and cause-specific mortality among patients with catatonia versus healthy‑matched controls, with ≥3 clinical diagnoses of catatonia

| Characteristics | Case, event (n = 5135) | Control, event (n = 20,540) | Crude hazard ratio (model 1) | Adjusted hazard ratio (model 2) |
| --- | --- | --- | --- | --- |
| All-cause | 1596 (31.1) | 2542 (12.4) | 2.88 (2.70–3.06)* | 2.55 (2.39–2.73)* |
| Natural causes | 1365 (26.6) | 2355 (11.5) | 2.66 (2.49–2.84)* | 2.36 (2.20–2.53)* |
| Certain infectious and parasitic diseases | 57 (1.1) | 78 (0.4) | 3.34 (2.38–4.71)* | 2.74 (1.92–3.90)* |
| Neoplasms | 243 (4.7) | 712 (3.5) | 1.57 (1.36–1.82)* | 1.31 (1.13–1.53)* |
| Diseases of the blood and blood-forming organs and certain disorders | 6 (0.1) | 8 (<0.1) | 3.32 (1.15-9.59)* | 3.13 (1.01-9.76)* |
| Endocrine, nutritional, and metabolic diseases | 126 (2.5) | 164 (0.8) | 3.52 (2.79–4.44)* | 3.07 (2.41–3.91)* |
| Mental and behavioral disorders | 58 (1.1) | 40 (0.2) | 6.73 (4.49–10.07)* | 6.14 (3.99–9.47)* |
| Diseases of the nervous system | 55 (1.1) | 36 (0.2) | 6.98 (4.58–10.62)* | 7.12 (4.55–11.14)* |
| Diseases of the eye and adnexa | 0 (0.0) | 0 (0.0) | – | – |
| Diseases of the ear and mastoid process | 0 (0.0) | 0 (0.0) | – | – |
| Diseases of the circulatory system | 300 (5.8) | 608 (3) | 2.27 (1.98–2.61)* | 2.15 (1.86–2.48)* |
| Diseases of the respiratory system | 222 (4.3) | 290 (1.4) | 3.49 (2.93–4.15)* | 3.18 (2.66–3.81)* |
| Diseases of the digestive system | 112 (2.2) | 156 (0.8) | 3.24 (2.54–4.13)* | 2.60 (2.02–3.35)* |
| Diseases of the skin and subcutaneous tissue | 9 (0.2) | 11 (0.1) | 3.72 (1.54–8.98)* | 3.29 (1.32–8.20)* |
| Diseases of the musculoskeletal system and connective tissue | 13 (0.3) | 23 (0.1) | 2.54 (1.29–5.02)* | 2.09 (1.03–4.22)* |
| Diseases of the genitourinary system | 82 (1.6) | 111 (0.5) | 3.36 (2.53–4.47)* | 2.97 (2.22–3.99)* |
| Pregnancy, childbirth, and the puerperium | 0 (0.0) | 0 (0.0) | – | – |
| Certain conditions originating in the perinatal period | 0 (0.0) | 0 (0.0) | – | – |
| Congenital malformations, deformations and chromosomal abnormalities | 2 (<0.1) | 1 (<0.1) | 9.62 (0.87-106.17) | 6.12 (0.45-83.49) |
| Symptoms, signs and abnormal clinical and laboratory findings, not elsewhere classified | 80 (1.6) | 116 (0.6) | 3.15 (2.37–4.18)* | 3.06 (2.26–4.15)* |
| Unnatural causes | 216 (4.2) | 166 (0.8) | 5.89 (4.81–7.21)* | 5.52 (4.43–6.89)* |
| Accident | 109 (2.1) | 90 (0.4) | 5.55 (4.20–7.34)* | 5.38 (3.96–7.31)* |
| Suicide | 105 (2.0) | 71 (0.3) | 6.62 (4.89–8.94)* | 6.17 (4.45–8.56)* |
| Assault / Homicide | 2 (<0.1) | 5 (<0.1) | 1.71 (0.33–8.82) | 0.94 (0.17–5.27) |
| Unknown causes | 15 (0.3) | 21 (0.1) | 3.46 (1.78–6.72)* | 3.93 (1.93–7.99)* |

^1^ Event was expressed as N (percentage).

^2^ Model 2 adjusted for all variables (birth year, sex, income level, urbanization level, and Charlson Comorbidity Index).

^3^ Asterisks indicate statistical significance.

**eTable 5.** The risk of all-cause and cause-specific mortality among patients with catatonia versus healthy‑matched controls, by age (adults)

| Characteristics | Case, event (n = 5539) | Control, event (n = 22,156) | Crude hazard ratio (model 1) | Adjusted hazard ratio (model 2) |
| --- | --- | --- | --- | --- |
| All-cause | 1268 (22.9) | 1347 (6.1) | 4.22 (3.91–4.55)* | 3.29 (3.03–3.57)* |
| Natural causes | 1011 (18.3) | 1166 (5.3) | 3.90 (3.58–4.24)* | 2.96 (2.70–3.24)* |
| Certain infectious and parasitic diseases | 40 (0.7) | 35 (0.2) | 5.09 (3.23–8.01)* | 3.00 (1.84–4.89)* |
| Neoplasms | 207 (3.7) | 488 (2.2) | 1.92 (1.63–2.25)* | 1.40 (1.18–1.67)* |
| Diseases of the blood and blood-forming organs and certain disorders | 4 (0.1) | 2 (<0.1) | 8.76 (1.60–47.85)* | 6.79 (1.13–40.98)* |
| Endocrine, nutritional, and metabolic diseases | 93 (1.7) | 66 (0.3) | 6.35 (4.63–8.70)* | 4.79 (3.42–6.70)* |
| Mental and behavioral disorders | 49 (0.9) | 14 (0.1) | 15.63 (8.63–28.31)* | 13.42 (7.08–25.45)* |
| Diseases of the nervous system | 51 (0.9) | 23 (0.1) | 9.89 (6.04–16.19)* | 9.14 (5.38–15.51)* |
| Diseases of the eye and adnexa | 0 (0.0) | 0 (0.0) | – | – |
| Diseases of the ear and mastoid process | 0 (0.0) | 0 (0.0) | – | – |
| Diseases of the circulatory system | 198 (3.6) | 263 (1.2) | 3.40 (2.83–4.09)* | 2.82 (2.31–3.43)* |
| Diseases of the respiratory system | 135 (2.4) | 58 (0.3) | 10.47 (7.70–14.24)* | 7.93 (5.73–10.97)* |
| Diseases of the digestive system | 104 (1.9) | 108 (0.5) | 4.26 (3.25–5.58)* | 2.90 (2.17–3.86)* |
| Diseases of the skin and subcutaneous tissue | 8 (0.1) | 6 (<0.1) | 6.09 (2.11–17.56)* | 4.58 (1.47–14.20)* |
| Diseases of the musculoskeletal system and connective tissue | 11 (0.2) | 14 (0.1) | 3.48 (1.58–7.68)* | 2.71 (1.15–6.35)* |
| Diseases of the genitourinary system | 48 (0.9) | 42 (0.2) | 5.12 (3.38–7.74)* | 3.95 (2.55–6.12)* |
| Pregnancy, childbirth, and the puerperium | 0 (0.0) | 1 (<0.1) | – | – |
| Certain conditions originating in the perinatal period | 0 (0.0) | 0 (0.0) | – | – |
| Congenital malformations, deformations and chromosomal abnormalities | 2 (<0.1) | 1 (<0.1) | 9.24 (0.84–101.94) | 6.09 (0.44–84.33) |
| Symptoms, signs and abnormal clinical and laboratory findings, not elsewhere classified | 61 (1.1) | 45 (0.2) | 6.00 (4.08–8.82)* | 5.31 (3.48–8.11)* |
| Unnatural causes | 246 (4.4) | 170 (0.8) | 6.36 (5.23–7.74)* | 5.91 (4.78–7.32)* |
| Accident | 118 (2.1) | 91 (0.4) | 5.77 (4.39–7.59)* | 5.31 (3.94–7.16)* |
| Suicide | 127 (2.3) | 74 (0.3) | 7.47 (5.61–9.95)* | 7.14 (5.23–9.74)* |
| Assault / Homicide | 1 (<0.1) | 5 (<0.1) | 0.84 (0.10–7.17) | 0.46 (0.05–4.23) |
| Unknown causes | 11 (0.2) | 11 (<0.1) | 4.81 (2.08–11.09)* | 4.59 (1.84–11.42)* |

^1^ Event was expressed as N (percentage).

^2^ Model 2 adjusted for all variables (birth year, sex, income level, urbanization level, and Charlson Comorbidity Index).

^3^ Asterisks indicate statistical significance.

**eTable 6.** The risk of all-cause and cause-specific mortality among patients with catatonia versus healthy‑matched controls, by age (older adults)

| Characteristics | Case, event (n = 1103) | Control, event (n = 4412) | Crude hazard ratio (model 1) | Adjusted hazard ratio (model 2) |
| --- | --- | --- | --- | --- |
| All-cause | 882 (80.0) | 2112 (47.9) | 2.89 (2.67–3.13)* | 2.06 (1.90–2.23)* |
| Natural causes | 845 (76.6) | 2048 (46.4) | 2.85 (2.63–3.09)* | 2.02 (1.86–2.19)* |
| Certain infectious and parasitic diseases | 40 (3.6) | 71 (1.6) | 3.99 (2.69–5.91)* | 2.61 (1.76–3.87)* |
| Neoplasms | 122 (11.1) | 463 (10.5) | 1.74 (1.42–2.13)* | 1.27 (1.03–1.55)* |
| Diseases of the blood and blood-forming organs and certain disorders | 3 (0.3) | 8 (0.2) | 2.23 (0.58–8.53) | 1.89 (0.48–7.45) |
| Endocrine, nutritional, and metabolic diseases | 70 (6.3) | 157 (3.6) | 2.99 (2.24–3.97)* | 2.15 (1.62–2.87)* |
| Mental and behavioral disorders | 27 (2.4) | 37 (0.8) | 5.85 (3.52–9.70)* | 4.03 (2.42–6.71)* |
| Diseases of the nervous system | 21 (1.9) | 26 (0.6) | 5.67 (3.16–10.17)* | 4.50 (2.49–8.11)* |
| Diseases of the eye and adnexa | 0 (0.0) | 0 (0.0) | – | – |
| Diseases of the ear and mastoid process | 0 (0.0) | 0 (0.0) | – | – |
| Diseases of the circulatory system | 219 (19.9) | 565 (12.8) | 2.77 (2.36–3.24)* | 1.96 (1.67–2.30)* |
| Diseases of the respiratory system | 171 (15.5) | 363 (8.2) | 3.25 (2.71–3.91)* | 2.24 (1.86–2.70)* |
| Diseases of the digestive system | 55 (5.0) | 88 (2.0) | 3.93 (2.79–5.53)* | 2.90 (2.06–4.09)* |
| Diseases of the skin and subcutaneous tissue | 4 (0.4) | 14 (0.3) | 1.81 (0.59–5.58) | 1.30 (0.42–4.04) |
| Diseases of the musculoskeletal system and connective tissue | 7 (0.6) | 15 (0.3) | 3.08 (1.23–7.67)* | 2.09 (0.84–5.20) |
| Diseases of the genitourinary system | 60 (5.4) | 124 (2.8) | 3.32 (2.43–4.54)* | 2.29 (1.67–3.13)* |
| Pregnancy, childbirth, and the puerperium | 0 (0.0) | 0 (0.0) | – | – |
| Certain conditions originating in the perinatal period | 0 (0.0) | 0 (0.0) | – | – |
| Congenital malformations, deformations and chromosomal abnormalities | 0 (0.0) | 1 (<0.1) | – | – |
| Symptoms, signs and abnormal clinical and laboratory findings, not elsewhere classified | 46 (4.2) | 116 (2.6) | 3.04 (2.15–4.30)* | 2.10 (1.48–2.99)* |
| Unnatural causes | 30 (2.7) | 48 (1.1) | 4.06 (2.55–6.46)* | 3.47 (2.16–5.58)* |
| Accident | 22 (2.0) | 36 (0.8) | 4.02 (2.34–6.90)* | 3.45 (1.98–6.02)* |
| Suicide | 7 (0.6) | 12 (0.3) | 3.68 (1.43–9.46)* | 3.14 (1.21–8.19)* |
| Assault / Homicide | 1 (0.1) | 0 (0.0) | – | – |
| Unknown causes | 7 (0.6) | 16 (0.4) | 4.04 (1.64–9.96)* | 3.34 (1.34–8.36)* |

^1^ Event was expressed as N (percentage).

^2^ Model 2 adjusted for all variables (birth year, sex, income level, urbanization level, and Charlson Comorbidity Index).

^3^ Asterisks indicate statistical significance.

**eTable 7.** The risk of all-cause and cause-specific mortality among patients with catatonia versus healthy‑matched controls, by sex (male)

| Characteristics | Case, event (n = 3744) | Control, event (n = 14,976) | Crude hazard ratio (model 1) | Adjusted hazard ratio (model 2) |
| --- | --- | --- | --- | --- |
| All-cause | 1296 (34.6) | 2039 (13.6) | 2.95 (2.76–3.17)* | 2.45 (2.28–2.64)* |
| Natural causes | 1107 (29.6) | 1874 (12.5) | 2.75 (2.55–2.96)* | 2.27 (2.10–2.45)* |
| Certain infectious and parasitic diseases | 49 (1.3) | 73 (0.5) | 3.12 (2.17–4.48)* | 2.29 (1.57–3.33)* |
| Neoplasms | 201 (5.4) | 599 (4.0) | 1.57 (1.34–1.84)* | 1.25 (1.06–1.47)* |
| Diseases of the blood and blood-forming organs and certain disorders | 5 (0.1) | 3 (<0.1) | 7.42 (1.77–31.08)* | 4.88 (1.11–21.49)* |
| Endocrine, nutritional, and metabolic diseases | 85 (2.3) | 103 (0.7) | 3.84 (2.88–5.12)* | 3.18 (2.36–4.29)* |
| Mental and behavioral disorders | 49 (1.3) | 26 (0.2) | 8.78 (5.45–14.12)* | 7.62 (4.55–12.75)* |
| Diseases of the nervous system | 48 (1.3) | 31 (0.2) | 7.24 (4.60–11.37)* | 6.41 (3.96–10.37)* |
| Diseases of the eye and adnexa | 0 (0.0) | 0 (0.0) | – | – |
| Diseases of the ear and mastoid process | 0 (0.0) | 0 (0.0) | – | – |
| Diseases of the circulatory system | 237 (6.3) | 455 (3.0) | 2.43 (2.08–2.85)* | 2.14 (1.82–2.52)* |
| Diseases of the respiratory system | 205 (5.5) | 259 (1.7) | 3.66 (3.05–4.40)* | 3.10 (2.56–3.74)* |
| Diseases of the digestive system | 103 (2.8) | 131 (0.9) | 3.61 (2.79–4.67)* | 2.56 (1.95–3.36)* |
| Diseases of the skin and subcutaneous tissue | 6 (0.2) | 7 (<0.1) | 4.07 (1.37–12.13)* | 3.57 (1.13–11.26)* |
| Diseases of the musculoskeletal system and connective tissue | 8 (0.2) | 15 (0.1) | 2.48 (1.05–5.84)* | 2.08 (0.86–5.05) |
| Diseases of the genitourinary system | 46 (1.2) | 77 (0.5) | 2.77 (1.92–3.99)* | 2.22 (1.52–3.23)* |
| Pregnancy, childbirth, and the puerperium | 0 (0.0) | 0 (0.0) | – | – |
| Certain conditions originating in the perinatal period | 0 (0.0) | 0 (0.0) | – | – |
| Congenital malformations, deformations and chromosomal abnormalities | 0 (0.0) | 0 (0.0) | – | – |
| Symptoms, signs and abnormal clinical and laboratory findings, not elsewhere classified | 65 (1.7) | 95 (0.6) | 3.16 (2.31–4.34)* | 3.05 (2.17–4.29)* |
| Unnatural causes | 178 (4.8) | 150 (1.0) | 5.47 (4.40–6.79)* | 5.11 (4.02–6.48)* |
| Accident | 96 (2.6) | 86 (0.6) | 5.19 (3.88–6.95)* | 4.95 (3.59–6.82)* |
| Suicide | 81 (2.2) | 60 (0.4) | 6.16 (4.41–8.60)* | 5.74 (3.99–8.27)* |
| Assault / Homicide | 1 (<0.1) | 4 (<0.1) | 1.10 (0.12–9.80) | 0.79 (0.08–7.75) |
| Unknown causes | 11 (0.3) | 15 (0.1) | 3.65 (1.67–7.95)* | 4.18 (1.81–9.66)* |

^1^ Event was expressed as N (percentage).

^2^ Model 2 adjusted for all variables (birth year, sex, income level, urbanization level, and Charlson Comorbidity Index).

^3^ Asterisks indicate statistical significance.

**eTable 8.** The risk of all-cause and cause-specific mortality among patients with catatonia versus healthy‑matched controls, by sex (female)

| Characteristics | Case, event (n = 2898) | Control, event (n = 11,592) | Crude hazard ratio (model 1) | Adjusted hazard ratio (model 2) |
| --- | --- | --- | --- | --- |
| All-cause | 854 (29.5) | 1420 (12.2) | 2.75 (2.53–3.00)* | 2.81 (2.58–3.07)* |
| Natural causes | 749 (25.8) | 1340 (11.6) | 2.56 (2.34–2.80)* | 2.63 (2.40–2.89)* |
| Certain infectious and parasitic diseases | 31 (1.1) | 33 (0.3) | 4.28 (2.62–7.00)* | 4.33 (2.60–7.20)* |
| Neoplasms | 128 (4.4) | 352 (3.0) | 1.67 (1.36–2.04)* | 1.55 (1.26–1.91)* |
| Diseases of the blood and blood-forming organs and certain disorders | 2 (0.1) | 7 (0.1) | 1.28 (0.27–6.16) | 1.78 (0.34–9.30) |
| Endocrine, nutritional, and metabolic diseases | 78 (2.7) | 120 (1.0) | 2.95 (2.22–3.93)* | 2.89 (2.16–3.88)* |
| Mental and behavioral disorders | 27 (0.9) | 25 (0.2) | 5.06 (2.93–8.72)* | 6.45 (3.61–11.52)* |
| Diseases of the nervous system | 24 (0.8) | 18 (0.2) | 6.09 (3.31–11.23)* | 7.35 (3.85–14.01)* |
| Diseases of the eye and adnexa | 0 (0.0) | 0 (0.0) | – | – |
| Diseases of the ear and mastoid process | 0 (0.0) | 0 (0.0) | – | – |
| Diseases of the circulatory system | 180 (6.2) | 373 (3.2) | 2.22 (1.86–2.65)* | 2.38 (1.98–2.87)* |
| Diseases of the respiratory system | 101 (3.5) | 162 (1.4) | 2.84 (2.21–3.64)* | 3.08 (2.38–3.97)* |
| Diseases of the digestive system | 56 (1.9) | 65 (0.6) | 3.90 (2.73–5.57)* | 3.93 (2.71–5.69)* |
| Diseases of the skin and subcutaneous tissue | 6 (0.2) | 13 (0.1) | 2.08 (0.79–5.48) | 2.12 (0.79–5.71) |
| Diseases of the musculoskeletal system and connective tissue | 10 (0.3) | 14 (0.1) | 3.20 (1.42–7.21)* | 2.42 (1.04–5.63)* |
| Diseases of the genitourinary system | 62 (2.1) | 89 (0.8) | 3.17 (2.29–4.38)* | 3.19 (2.29–4.45)* |
| Pregnancy, childbirth, and the puerperium | 0 (0.0) | 1 (<0.1) | – | – |
| Certain conditions originating in the perinatal period | 0 (0.0) | 0 (0.0) | – | – |
| Congenital malformations, deformations and chromosomal abnormalities | 2 (0.1) | 2 (<0.1) | 4.75 (0.67–33.72) | 4.09 (0.51–32.87) |
| Symptoms, signs and abnormal clinical and laboratory findings, not elsewhere classified | 42 (1.4) | 66 (0.6) | 2.96 (2.01–4.36)* | 3.18 (2.12–4.76)* |
| Unnatural causes | 98 (3.4) | 68 (0.6) | 6.52 (4.79–8.89)* | 6.46 (4.61–9.04)* |
| Accident | 44 (1.5) | 41 (0.4) | 4.94 (3.22–7.56)* | 5.01 (3.16–7.96)* |
| Suicide | 53 (1.8) | 26 (0.2) | 9.09 (5.68–14.53)* | 9.09 (5.48–15.10)* |
| Assault / Homicide | 1 (<0.1) | 1 (<0.1) | 4.18 (0.26–66.78) | 1.29 (0.07–24.01) |
| Unknown causes | 7 (0.2) | 12 (0.1) | 2.82 (1.11–7.18)* | 2.95 (1.09–7.93)* |

^1^ Event was expressed as N (percentage).

^2^ Model 2 adjusted for all variables (birth year, sex, income level, urbanization level, and Charlson Comorbidity Index).

^3^ Asterisks indicate statistical significance.

**eTable 9.** The risk of all-cause and cause-specific mortality among patients with catatonia versus healthy‑matched controls, by etiology (psychosis‑related)

| Characteristics | Case, event (n = 4169) | Control, event (n =16,676) | Crude hazard ratio (model 1) | Adjusted hazard ratio (model 2) |
| --- | --- | --- | --- | --- |
| All-cause | 954 (22.9) | 1308 (7.8) | 3.22 (2.97–3.51)* | 2.73 (2.49–2.99)* |
| Natural causes | 771 (18.5) | 1166 (7.0) | 2.93 (2.67–3.21)* | 2.47 (2.24–2.72)* |
| Certain infectious and parasitic diseases | 36 (0.9) | 40 (0.2) | 3.95 (2.52–6.20)* | 2.89 (1.78–4.69)* |
| Neoplasms | 138 (3.3) | 425 (2.5) | 1.44 (1.19–1.75)* | 1.16 (0.95–1.43) |
| Diseases of the blood and blood-forming organs and certain disorders | 4 (0.1) | 5 (<0.1) | 3.37 (0.91–12.56) | 2.94 (0.73–11.91) |
| Endocrine, nutritional, and metabolic diseases | 75 (1.8) | 79 (0.5) | 4.20 (3.06–5.76)* | 3.58 (2.55–5.01)* |
| Mental and behavioral disorders | 46 (1.1) | 17 (0.1) | 11.94 (6.85–20.83)* | 10.76 (5.87–19.73)* |
| Diseases of the nervous system | 28 (0.7) | 21 (0.1) | 5.86 (3.33–10.33)* | 5.17 (2.76–9.68)* |
| Diseases of the eye and adnexa | 0 (0.0) | 0 (0.0) | – | – |
| Diseases of the ear and mastoid process | 0 (0.0) | 0 (0.0) | – | – |
| Diseases of the circulatory system | 167 (4.0) | 277 (1.7) | 2.68 (2.22–3.25)* | 2.47 (2.01–3.04)* |
| Diseases of the respiratory system | 116 (2.8) | 98 (0.6) | 5.22 (3.99–6.83)* | 4.51 (3.39–5.99)* |
| Diseases of the digestive system | 67 (1.6) | 87 (0.5) | 3.37 (2.45–4.63)* | 2.20 (1.57–3.09)* |
| Diseases of the skin and subcutaneous tissue | 6 (0.1) | 4 (<0.1) | 6.53 (1.84–23.15)* | 4.67 (1.22–17.87)* |
| Diseases of the musculoskeletal system and connective tissue | 6 (0.1) | 11 (0.1) | 2.39 (0.88–6.47) | 2.00 (0.67–6.01) |
| Diseases of the genitourinary system | 32 (0.8) | 50 (0.3) | 2.83 (1.81–4.40)* | 2.65 (1.67–4.22)* |
| Pregnancy, childbirth, and the puerperium | 0 (0.0) | 0 (0.0) | – | – |
| Certain conditions originating in the perinatal period | 0 (0.0) | 0 (0.0) | – | – |
| Congenital malformations, deformations and chromosomal abnormalities | 2 (0.0) | 1 (0.0) | 9.09 (0.82–100.29) | 6.36 (0.46–88.51) |
| Symptoms, signs and abnormal clinical and laboratory findings, not elsewhere classified | 48 (1.2) | 51 (0.3) | 4.17 (2.81–6.18)* | 4.18 (2.71–6.47)* |
| Unnatural causes | 178 (4.3) | 135 (0.8) | 5.76 (4.61–7.21)* | 5.44 (4.25–6.96)* |
| Accident | 79 (1.9) | 75 (0.4) | 4.66 (3.40–6.39)* | 4.47 (3.15–6.34)* |
| Suicide | 98 (2.4) | 58 (0.3) | 7.30 (5.28–10.11)* | 6.98 (4.89–9.95)* |
| Assault / Homicide | 1 (<0.1) | 2 (<0.1) | 2.08 (0.19–22.91) | 0.63 (0.05–7.59) |
| Unknown causes | 5 (0.1) | 7 (<0.1) | 3.36 (1.06–10.59)* | 3.58 (1.04–12.31)* |

^1^ Event was expressed as N (percentage).

^2^ Model 2 adjusted for all variables (birth year, sex, income level, urbanization level, and Charlson Comorbidity Index).

^3^ Asterisks indicate statistical significance.

**eTable 10.** The risk of all-cause and cause-specific mortality among patients with catatonia versus healthy‑matched controls, by etiology (non‑psychosis‑related)

| Characteristics | Case, event (n = 2473) | Control, event (n = 9892) | Crude hazard ratio (model 1) | Adjusted hazard ratio (model 2) |
| --- | --- | --- | --- | --- |
| All-cause | 1196 (48.4) | 2151 (21.7) | 2.83 (2.64–3.04)* | 2.48 (2.31–2.67)* |
| Natural causes | 1085 (43.9) | 2048 (20.7) | 2.70 (2.51–2.91)* | 2.36 (2.19–2.55)* |
| Certain infectious and parasitic diseases | 44 (1.8) | 66 (0.7) | 3.41 (2.32–4.99)* | 2.86 (1.93–4.23)* |
| Neoplasms | 191 (7.7) | 526 (5.3) | 1.85 (1.57–2.18)* | 1.50 (1.26–1.77)* |
| Diseases of the blood and blood-forming organs and certain disorders | 3 (0.1) | 5 (0.1) | 3.07 (0.73–12.9) | 3.45 (0.76–15.76) |
| Endocrine, nutritional, and metabolic diseases | 88 (3.6) | 144 (1.5) | 3.08 (2.36–4.01)* | 2.63 (2.01–3.45)* |
| Mental and behavioral disorders | 30 (1.2) | 34 (0.3) | 4.64 (2.84–7.59)* | 4.80 (2.87–8.02)* |
| Diseases of the nervous system | 44 (1.8) | 28 (0.3) | 8.12 (5.05–13.06)* | 8.06 (4.9–13.26)* |
| Diseases of the eye and adnexa | 0 (0.0) | 0 (0.0) | – | – |
| Diseases of the ear and mastoid process | 0 (0.0) | 0 (0.0) | – | – |
| Diseases of the circulatory system | 250 (10.1) | 551 (5.6) | 2.33 (2.01–2.71)* | 2.12 (1.82–2.47)* |
| Diseases of the respiratory system | 190 (7.7) | 323 (3.3) | 2.99 (2.50–3.58)* | 2.63 (2.19–3.16)* |
| Diseases of the digestive system | 92 (3.7) | 109 (1.1) | 4.20 (3.18–5.55)* | 3.53 (2.65–4.69)* |
| Diseases of the skin and subcutaneous tissue | 6 (0.2) | 16 (0.2) | 1.94 (0.76–4.96) | 2.07 (0.79–5.45) |
| Diseases of the musculoskeletal system and connective tissue | 12 (0.5) | 18 (0.2) | 3.35 (1.61–6.96)* | 2.68 (1.28–5.65)* |
| Diseases of the genitourinary system | 76 (3.1) | 116 (1.2) | 3.32 (2.48–4.43)* | 2.82 (2.1–3.79)* |
| Pregnancy, childbirth, and the puerperium | 0 (0.0) | 1 (<0.1) | – | – |
| Certain conditions originating in the perinatal period | 0 (0.0) | 0 (0.0) | – | – |
| Congenital malformations, deformations and chromosomal abnormalities | 0 (0.0) | 1 (<0.1) | – | – |
| Symptoms, signs and abnormal clinical and laboratory findings, not elsewhere classified | 59 (2.4) | 110 (1.1) | 2.75 (2.00–3.78)* | 2.65 (1.91–3.68)* |
| Unnatural causes | 98 (4.0) | 83 (0.8) | 5.86 (4.37–7.85)* | 5.47 (3.99–7.50)* |
| Accident | 61 (2.5) | 52 (0.5) | 5.90 (4.07–8.55)* | 5.76 (3.85–8.62)* |
| Suicide | 36 (1.5) | 28 (0.3) | 6.26 (3.81–10.26)* | 5.47 (3.23–9.26)* |
| Assault / Homicide | 1 (<0.1) | 3 (<0.1) | 1.56 (0.16–14.98) | 1.52 (0.14–16.31) |
| Unknown causes | 13 (0.5) | 20 (0.2) | 3.66 (1.82–7.36)* | 3.49 (1.67–7.27)* |

^1^ Event was expressed as N (percentage).

^2^ Model 2 adjusted for all variables (birth year, sex, income level, urbanization level, and Charlson Comorbidity Index).

^3^ Asterisks indicate statistical significance.

**Table 11.** The risk of all-cause and cause-specific mortality among patients with catatonia versus their unaffected siblings

| Characteristics | Crude hazard ratio (model 1) |
| --- | --- |
| All-cause | 2.97 (2.31–3.82)* |
| Natural causes | 2.94 (2.16–3.99)* |
| Certain infectious and parasitic diseases | 8.16 (0.94–70.67) |
| Neoplasms | 2.61 (1.25–5.47)* |
| Diseases of the blood and blood-forming organs and certain disorders | – |
| Endocrine, nutritional, and metabolic diseases | 2.06 (0.55–7.69) |
| Mental and behavioral disorders | 19.77 (2.58–151.74)* |
| Diseases of the nervous system | 1.63 (0.57–4.65) |
| Diseases of the eye and adnexa | – |
| Diseases of the ear and mastoid process | – |
| Diseases of the circulatory system | 3.04 (1.57–5.87)* |
| Diseases of the respiratory system | 4.38 (1.71–11.20)* |
| Diseases of the digestive system | 3.00 (1.11–8.14)* |
| Diseases of the skin and subcutaneous tissue | 1.63 (0.10–27.11) |
| Diseases of the musculoskeletal system and connective tissue | 4.88 (0.52–46.02) |
| Diseases of the genitourinary system | 0.82 (0.07–8.97) |
| Pregnancy, childbirth, and the puerperium | – |
| Certain conditions originating in the perinatal period | – |
| Congenital malformations, deformations and chromosomal abnormalities | – |
| Symptoms, signs and abnormal clinical and laboratory findings, not elsewhere classified | 1.63 (0.57–4.64) |
| Unnatural causes | 3.03 (1.89–4.83)* |
| Accident | 3.42 (1.66–7.04)* |
| Suicide | 2.63 (1.40–4.94)* |
| Assault / Homicide | – |
| Unknown causes | 3.39 (0.31–37.24) |

^1^ Asterisks indicate statistical significance.

**Table 12.** The risk of all-cause and cause-specific mortality among patients with schizophrenia spectrum disorders with versus without catatonia

| Characteristics | Crude hazard ratio (model 1) |
| --- | --- |
| All-cause | 0.95 (0.89–1.01) |
| Natural causes | 0.94 (0.87–1.00) |
| Certain infectious and parasitic diseases | 1.23 (0.89–1.71) |
| Neoplasms | 0.97 (0.82–1.14) |
| Diseases of the blood and blood-forming organs and certain disorders | 1.39 (0.52–3.75) |
| Endocrine, nutritional, and metabolic diseases | 1.01 (0.81–1.27) |
| Mental and behavioral disorders | 1.28 (0.95–1.71) |
| Diseases of the nervous system | 1.15 (0.79–1.68) |
| Diseases of the eye and adnexa | – |
| Diseases of the ear and mastoid process | – |
| Diseases of the circulatory system | 0.82 (0.70–0.95)* |
| Diseases of the respiratory system | 0.86 (0.71–1.03) |
| Diseases of the digestive system | 1.01 (0.79–1.28) |
| Diseases of the skin and subcutaneous tissue | 1.29 (0.58–2.89) |
| Diseases of the musculoskeletal system and connective tissue | 0.90 (0.40–2.00) |
| Diseases of the genitourinary system | 0.72 (0.51–1.02) |
| Pregnancy, childbirth, and the puerperium | – |
| Certain conditions originating in the perinatal period | – |
| Congenital malformations, deformations and chromosomal abnormalities | 2.77 (0.68–11.38) |
| Symptoms, signs and abnormal clinical and laboratory findings, not elsewhere classified | 0.88 (0.66–1.17) |
| Unnatural causes | 1.01 (0.87–1.17) |
| Accident | 1.28 (1.02–1.60)* |
| Suicide | 0.87 (0.71–1.06) |
| Assault / Homicide | 0.58 (0.08–4.18) |
| Unknown causes | 0.78 (0.32–1.88) |

^1^ Asterisks indicate statistical significance.

**eFigure 1.** Flowchart of the selection process for the study

**
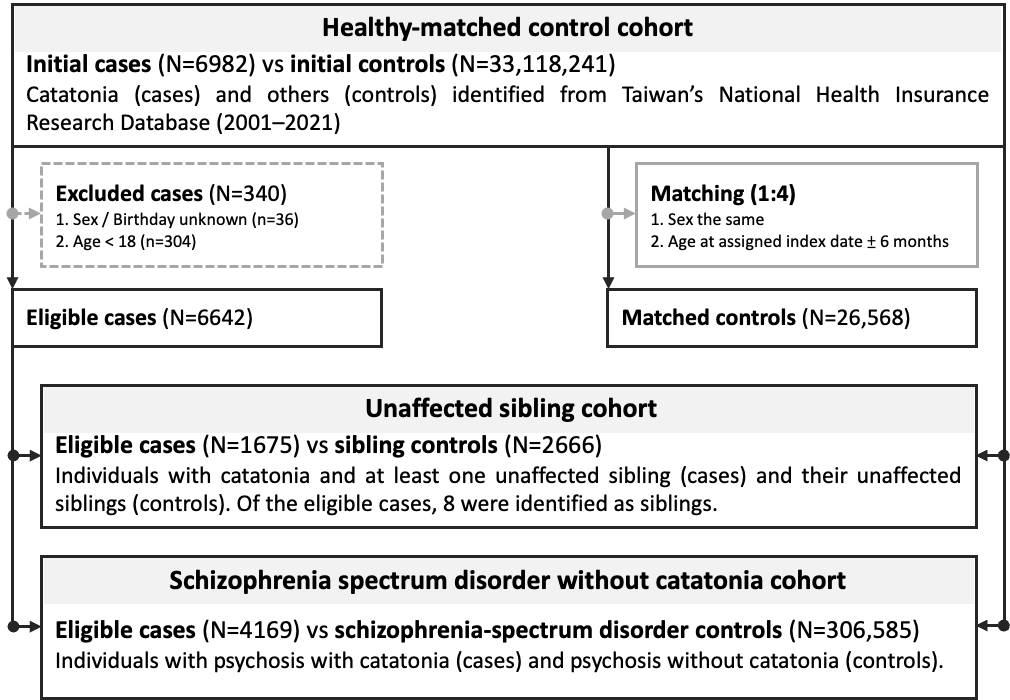
**
